# Analysis of HER2 Expression in Different Histological Subtypes and IHC-Based Molecular Variants of Muscle-Invasive Bladder Carcinoma

**DOI:** 10.3390/medicina61101759

**Published:** 2025-09-28

**Authors:** Elitsa Kraevska, Savelina Popovska

**Affiliations:** 1 Department of Pathoanatomy, Faculty of Medicine, Medical University-Pleven, 5800 Pleven, Bulgaria; 2 Centre of Competence in Personalized Medicine, 3D and Telemedicine, Robotic Assisted and Minimally Invasive Surgery-“Leonardo da Vinci” Pleven, 5800 Pleven, Bulgaria

**Keywords:** muscle-invasive bladder carcinoma, MIBC, HER2, immunohistochemistry, histological subtypes, molecular variants, targeted therapy

## Abstract

*Background and Objectives*: Urothelial carcinoma of the urinary bladder is a heterogeneous disease with diverse morphological and molecular characteristics. This study aims to analyze the expression of HER2 in 100 consecutive cases of muscle-invasive bladder carcinoma (MIBC), with a special attention to the different histological subtypes and consensus molecular variants determined by IHC methods. *Materials and Methods*: A retrospective single-center study was conducted on 100 consecutive cases of MIBC (2021–2024). HER2 status is assessed by immunohistochemistry (IHC) (scores 0, 0+, 1+, 2+, 3+), and the results are compared with the published data. *Results*: We have established that over half of the tumors (~60%) show some level of HER2 expression, with strong expression (3+) present in 25%. There are significant differences among the IHC-based molecular variants: luminal tumors, including papillary tumors, exhibit a frequent HER2 overexpression, whereas those with a basal immunophenotype (e.g., squamous, sarcomatoid variants) are almost entirely HER2-negative. The micropapillary subtype and some other rare subtypes can also express HER2. *Conclusions*: HER2 is an important biomarker with heterogeneous expression in urothelial carcinoma of the bladder. The present study showed that the frequency and level of HER2 expression vary substantially among different histopathological subtypes and molecular variants. In therapeutic terms, interest in HER2 as a target is growing—new antibody–drug conjugates show a promising activity even in cases with low HER2 expression, which will likely lead to the integration of HER2-directed therapies and routine testing in the future.

## 1. Introduction

Bladder carcinoma (BC) is one of the most common malignancies, ranking 9th globally, and according to GLOBOCAN there were 614,298 new BC cases and 220,596 deaths worldwide in 2022 [1]. The disease predominantly affects males (approximately 3–4 times more often than females) and usually occurs at an advanced age [2]. Over 90% of bladder carcinomas in developed countries are urothelial carcinomas. The remaining <10% are other histological types—most commonly squamous cell carcinomas and less often adenocarcinomas [2]. According to the WHO, urothelial cancers are classified by grade into low-grade and high-grade, with carcinoma in situ (CIS) considered separately as a flat high-grade tumor. Approximately 25–30% of bladder cancer cases are muscle-invasive (stage T2 or higher)—for these, the standard treatment is radical cystectomy with or without neoadjuvant/adjuvant therapy [2]. Muscle-invasive urothelial carcinoma of the bladder (MIBC) is associated with a less favorable prognosis compared to superficial forms of the disease, even with multimodal treatment.

Human Epidermal Growth Factor Receptor 2 (HER2, also known as ERBB2) is an oncogene encoding a transmembrane receptor with tyrosine kinase activity. It is an established prognostic and predictive biomarker in breast and gastric cancer, where HER2 overexpression is associated with a more aggressive disease and serves as a target for targeted therapy. In the context of bladder carcinoma, data on the role of HER2 are conflicting. Some studies report a low frequency of HER2-positivity (~6–15%) in advanced urothelial carcinoma, while others indicate higher values (up to 30% or more) in certain cases. In recent years, interest in HER2 in bladder cancer has increased due to the development of new therapies, including monoclonal antibodies and antibody–drug conjugates (ADCs) targeting HER2. Different authors have proposed various criteria for evaluating HER2 expression in urothelial carcinomas.

The present study summarizes the current data on the frequency of HER2 expression in MIBC, including the distribution of cases by IHC scores of 0, 0+, 1+, 2+, and 3+. Special emphasis is placed on HER2 expression in the different histological subtypes and IHC-based molecular variants of MIBC, as well as the significance of HER2 as a diagnostic, prognostic, and therapeutic factor. The literature data are compared with the results of our own retrospective cohort study of 100 consecutive patients, conducted in the period 2021–2024, with the aim of gaining a deeper understanding of the role of HER2 in this disease.

## 2. Materials and Methods

### 2.1. Study Design and Clinical Cohort

A retrospective single-center study was conducted among patients with MIBC treated in the Urology Clinic of University Hospital “Sveta Marina”—Pleven, Bulgaria, over 4 years (January 2021–December 2024). One hundred consecutive patients with histologically confirmed muscle-invasive urothelial carcinoma of the bladder who underwent radical cystectomy (RC) or transurethral resection (TURB) of the tumor were included. Inclusion criteria required invasion of the tumor into the detrusor muscle (stage T2 or higher) in the submitted biopsy or surgical specimen. Exclusion criteria were non-muscle-invasive tumors (pTa, pT1), a repeat biopsy of the same patient, and insufficient tissue material at risk of depletion. Over 1000 histological slides from the archive were reviewed for cohort selection, of which these 100 met the specified criteria. Necessary clinical data (sex, age) were extracted from electronic medical records, discharge summaries, and pathology reports. Staging was performed according to the TNM classification of the World Health Organization (WHO, 5th revision, 2022) [3]. Tumors were grouped by histopathological subtypes (according to the WHO 2022 classification) and by surrogate IHC-based molecular subtypes (according to the consensus molecular classification of MIBC, 2020 [4]) for the analysis.

### 2.2. Patient Characteristics

Among the 100 patients, 86% were male and 14% female, which corresponds to the known gender distribution in bladder cancer. The median age was 69.5 years (interquartile range 64–75 years), and 90% of the patients were over 55 years old at the time of diagnosis, consistent with the epidemiology of the disease. In 66% of patients, TURB of the tumor was performed, while the remaining 34% underwent radical cystectomy.

### 2.3. Histological and Molecular Subtyping of Tumors

All carcinomas in the cohort were high-grade, and variant morphology was frequently observed. The largest subset was papillary invasive carcinoma—27% of cases. A significant proportion of tumors (27/100) had a mixed histological composition, i.e., a combination of two or more subtype components. The frequency of the main histological subtype in the cohort is shown in Table 1 (column “n”). In summary, aside from 4% urothelial carcinomas with no specific features (NOS), the cohort included 17% urothelial carcinoma with squamous differentiation, 5% sarcomatoid, 4% micropapillary, 3% urothelial carcinoma with glandular differentiation, 3% plasmacytoid, 2% nested, and 5% small cell carcinomas. Separately, an IHC-based molecular stratification of the same cases, performed in a previous analysis (Table 2), showed that 50% of the tumors fall into the luminal variants (including Luminal Papillary (LumP), Luminal Unstable (LumU), and Luminal Non-Specified (Lum NS)), 33% are Basal/Squamous, 9% are basal–luminal (double-positive), and the remaining 8% are double-negative (6% neuroendocrine-like and 2% stroma-rich).

### 2.4. Immunohistochemical Study of HER2

HER2 expression was evaluated by the immunohistochemistry method (IHC). The slides were evaluated independently by two pathologists—one professor (SLP) and one uropathologist (EPK)—and, in cases of disagreement, results were evaluated by consensus. All tissues were fixed in neutral formalin and embedded in paraffin according to standard protocols. Sections 4 μm thick were cut from selected blocks and stained with the HercepTest™ kit (Dako, Glostrup, Denmark/Agilent, Santa Clara, CA, USA)—a standardized system routinely used for HER2 testing in breast cancer. The staining employed a polyclonal rabbit anti-HER2 antibody (code A0485) directed against the intracellular domain of the HER2 receptor. An automated platform (Dako Autostainer Link 48 with PT Link) was used, strictly following the manufacturer’s instructions. Heat-induced epitope retrieval (HIER) was performed in a low pH buffer, followed by incubation with the primary antibody for 20–30 min and visualization via an HRP-polymer system with DAB as the chromogen. Internal control samples were included in each staining run, including a provided multi-control slide (four breast cancer cell lines with expression levels 0, 1+, 2+, 3+) and additional in-house negative controls from tumor tissue.

### 2.5. Interpretation of HER2 Status

The IHC staining results for HER2 are interpreted in accordance with the latest ASCO/CAP criteria (2025) [5], analogous to those for breast carcinoma. Five main categories were defined: 0 (complete absence of specific membranous staining); 0+ (weak, incomplete staining in ≤10% of invasive tumor cells—this includes the “ultra low” group, which shows minimal expression in ≤ 10% of invasive tumor cells), 1+ (weak, incomplete membranous staining in >10% of invasive tumor cells), 2+ (weak to moderately intense, complete membranous staining in >10% of invasive tumor cells), and 3+ (strong, complete membranous staining in >10% of invasive tumor cells). In accordance with clinical standards, a HER2-positive tumor is defined by a 3+ result (overexpression) or 2+ with confirmed gene amplification (by FISH/SISH). In our study, additional in situ hybridization was not performed; 2+ cases are considered to have moderate expression (borderline status). Tumors with an IHC score of 0 are considered HER2-negative. HER2-low expressing tumors are those with 1+. Special attention is given to the so-called “HER2 ultra-low” category 0+—minimal immunoreactivity (barely perceptible faint membranous staining) in ≤10% of invasive tumor cells. Although unified criteria for “ultra-low” in urothelial carcinomas are lacking, we recorded such cases separately in our cohort, as this threshold has potential research and therapeutic relevance.

### 2.6. Statistical Analysis

Data processing was performed using Statistical Package for the Social Sciences v.25 (SPSS Inc., Chicago, IL, USA). Statistical analysis included descriptive methods for frequency distribution and percentages, with calculation of mean, median, standard deviation, and range for quantitative measures. χ^2^ tests were used for comparison of proportions, with *p* < 0.05 considered statistically significant.

### 2.7. Ethical Aspects

The study was conducted following the national and international requirements for clinical studies, including the preservation of the anonymity of the participants and the non-disclosure of their personal information. Each participant signed an informed consent form during their hospital stay. The study was conducted in accordance with the requirements of the Ethics Committee of the Medical University of Pleven, Bulgaria: approval number № 781/14 June 2024.

## 3. Results

Of the 100 carcinomas studied, 25% exhibited strong HER2 expression (IHC score 3+), and an additional 22% were 2+ (moderate expression)—i.e., a total of 47% are potentially HER2-positive. The remaining cases are distributed as follows: 13% weakly positive (1+), 9% (0+) “ultra-low” (barely perceptible staining in ≤10% of cells), and 31% completely negative (0) (Table 3). The data show that over half of the tumors (~60%) demonstrate some level of membranous HER2 reactivity, although cases with strong expression (3+) constitute one quarter of the total.

In our series, HER2-overexpressing tumors (IHC 3+) are observed in approximately the same proportion (25–30%) as reported in some more recent studies. At the same time, the proportion of HER2-low cases (1+ or 2+ without amplification)—around 35% of all cases—is higher than that in certain previous series of advanced bladder cancer (~17–20% in the literature) [6], but, in our study, additional in situ hybridization was not performed, so some of the 2+ cases may fall into the HER2-positive group. Our study on the HER2 2+ category will continue using in situ hybridization methods to determine exactly what percentage of these tumors are positive. A small proportion (9%) of 0+ (“ultra-low”) cases is also noted, a category which still has no established clinical significance but is gaining interest in the context of new therapies.

## 4. Discussion

### 4.1. HER2 Expression and Molecular Variant

The contemporary consensus molecular classification of MIBC defines six main variants—Luminal Papillary (LumP), Luminal Non-Specified (LumNS), Luminal Unstable (LumU), Basal/Squamous, stroma-rich, and neuroendocrine-like (NE-like) [4]. Each variant has distinct molecular and phenotypic features, and one of the key differences is the HER2 profile. Luminal tumors (especially Luminal Papillary and Luminal Unstable) often demonstrate HER2 overexpression, whereas Basal/Squamous tumors usually do not express HER2 to a significant degree [7]. Our results confirm this trend (Table 2).

For the Basal/Squamous variant, no cases with 3+ were observed, and only a few tumors (~3%) showed 2+; the rest were 0 or 1+, including approximately 18% with an “ultra-low” minimal reaction. As shown, the luminal carcinomas have a significantly higher frequency of HER2 expression. Luminal Papillary tumors are 3+ in 33% of cases and 2+ in 38%, i.e., ~71% have at least moderate expression. Luminal Unstable tumors show an even higher proportion—56% with 3+ and 31% with 2+ (87% overall with expression). The LumNS variant is also frequently HER2-positive (50% 3+ and 30% 2+). In contrast, in the Basal/Squamous variant, no cases of 3+ overexpression were observed, and only isolated tumors showed 2+; the majority are completely negative or weakly positive (0/1+). The BasoLuminal (double-positive) tumors occupy an intermediate position—around 33% 3+ and 22% 2+—with the remainder predominantly 1+. The two variants (NE-like and stroma-rich) do not exhibit HER2 overexpression—in our cohort, all neuroendocrine-like cases (n = 6) are 0, and of the two stroma-rich cases one is 0 and one is “ultra-low.” These data highlight that HER2 status may be related to the tumor’s molecular profile: luminal carcinomas express HER2 significantly more often compared to basal carcinomas (*p* < 0.001). In practice, strong circumferential membranous HER2 reactivity can serve as an indirect marker pointing to a luminal phenotype, whereas a lack of HER2 is more characteristic of basal and double-negative tumors.

### 4.2. HER2 Expression and Histological Subtype

Urothelial carcinoma exhibits numerous distinctive histopathological subtypes (WHO 2022), which are often associated with aggressive biological behavior. In our series, we observe that different subtypes have different HER2 profiles (Table 3).

**Figure 1:**
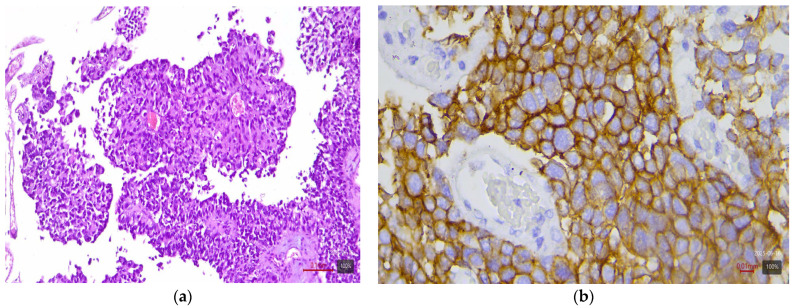
Papillary urothelial carcinoma (LumP molecular variant) with HER2 3+ expression: (**a**) H&E; (**b**) HER2.

**Figure 2:**
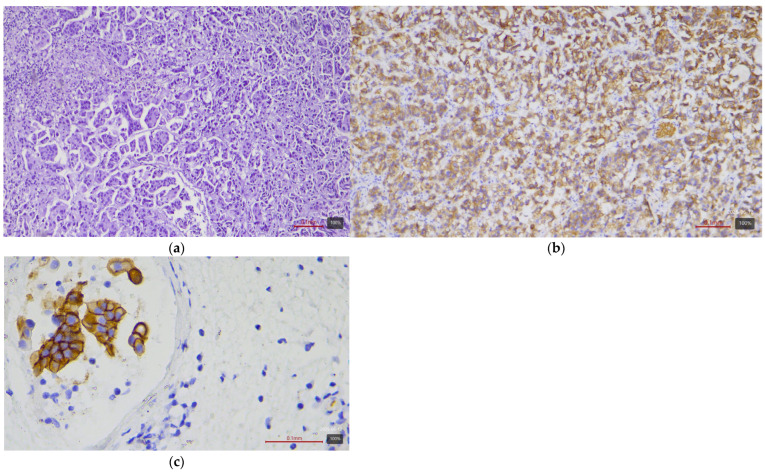
Micropapillary subtype urothelial carcinoma (LumU molecular variant) with HER2 3+ expression: (**a**) H&E; (**b**,**c**) HER2.

From the table, it can be seen that papillary and micropapillary carcinomas—which have a luminal phenotype—are enriched in HER2-positive cases. Invasive papillary tumors (Figure 1) show 3+ in 44% and 2+ in 26% of cases (~70% with any expression). The micropapillary subtype (Figure 2) is even more pronounced in this respect: in our cohort, three out of four cases (75%) are HER2 3+, and one is 2+. This aggressive subtype of urothelial carcinoma is known for frequent HER2 activation—the literature sources report up to ~30–40% HER2-positivity by IHC. For the plasmacytoid subtype (a rare discohesive tumor with a CDH1 mutation), data are limited, but small studies also find relatively high HER2 expression (up to 83% with positive/borderline status in one series of six cases by Kim et al. [8]). The three plasmacytoid carcinomas from our cohort are evenly distributed: one each with 1+, 2+, and 3+ (33% in each category). This confirms that even this subtype can express HER2, albeit inconsistently.

**Figure 3:**
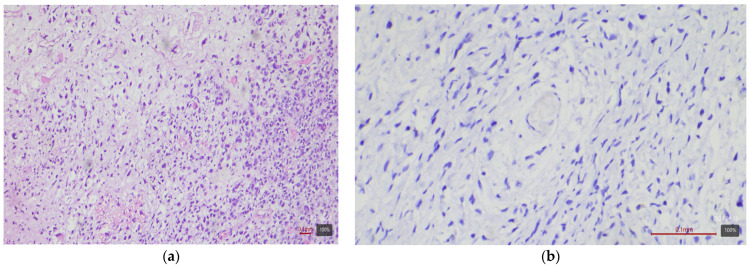
Sarcomatoid urothelial carcinoma (Stroma rich molecular variant) with HER2 0 expression: (**a**) H&E; (**b**) HER2.

**Figure 4:**
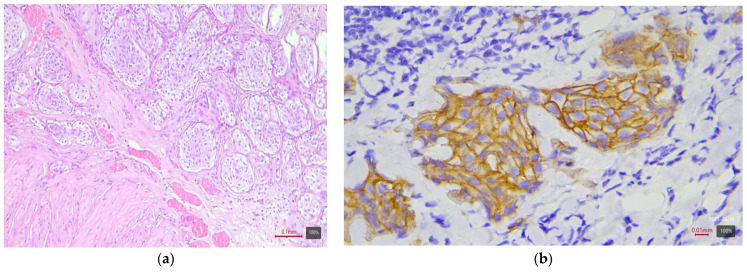
Nested urothelial carcinoma (LumP molecular variant) with HER2 3+ expression: (**a**) H&E; (**b**) HER2.

**Figure 5:**
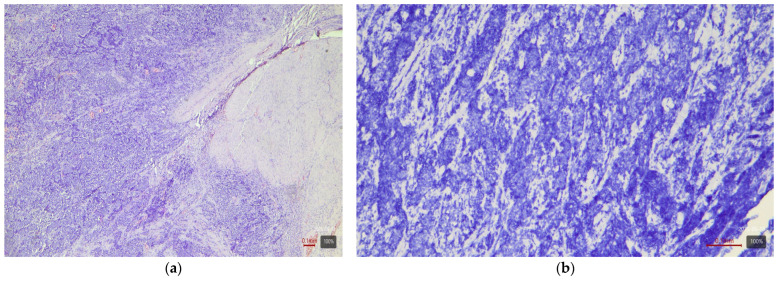
Small cell carcinoma (NE-like molecular variant) with HER2 0 expression: (**a**) H&E; (**b**) HER2.

Subtypes with squamous or sarcomatoid differentiation—which fall into the basal molecular class—showed no HER2 overexpression (Figure 3). Among the 17 tumors with areas of squamous differentiation in our study, none is 2+ or 3+; nearly half (47%) are completely negative, and the rest are weakly positive (mostly 1+ or 0+). Similarly, of five sarcomatoid carcinomas, none exhibited HER2 2+ or 3+ (all are 0/1+). The nested variant showed opposite results in our two cases—one is 3+ (Figure 4), the other 0—so it is difficult to draw conclusions from this small number of cases. The small cell carcinoma, as expected, did not express HER2—all five cases in the cohort are completely negative (Figure 5).

**Figure 6:**
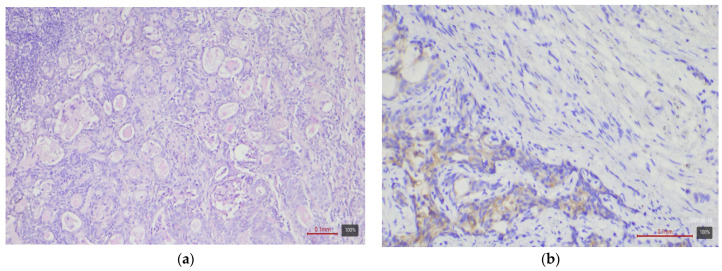
Microcystic subtype with HER2 1+ expression: (**a**) H&E; (**b**) HER2.

**Figure 7:**
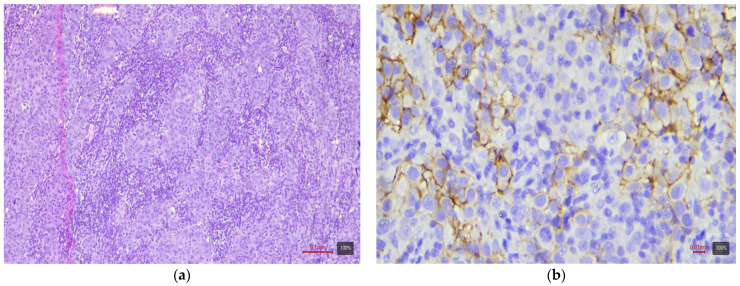
Lymphoepithelioma-like UC with HER2 2+ expression: (**a**) H&E; (**b**) HER2.

Some of the tumors have additional rare morphological components—e.g., microcystic, giant cell, or lymphoepithelioma-like features. These variants are present only in combined (mixed) tumors. In the individual cases with such differentiation, we observed varied results: for example, a tumor with a microcystic component is HER2 1+ (Figure 6), while a tumor with a lymphoepithelioma-like component showed 2+ expression (Figure 7). Due to the very small numbers, these observations are descriptive. However, overall, they reinforce the trend that histological subtypes with a luminal phenotype (papillary, micropapillary) are associated with increased HER2 expression, whereas those with basal differentiation (squamous, sarcomatoid) are HER2-negative. The plasmacytoid and some other rare subtypes can also be HER2-expressing.

### 4.3. Diagnostic Significance of HER2 in Bladder Carcinoma

In the routine diagnosis of urothelial carcinomas, testing for HER2 is not a standard practice, unlike in breast or gastric cancer. The histopathological diagnosis and classification of the tumor are based mainly on morphological features and classic immunohistochemical markers (e.g., p63, GATA3, CK5/6, CK20, etc.), while HER2 is not used. Nevertheless, the results of our study and the literature indicate that HER2 expression can have a useful additional diagnostic role in certain situations. For example, strong and circumferential membranous HER2 reactivity in small papillary structures may alert the pathologist to a micropapillary subtype of the tumor, especially when the morphology is not definitive. In such cases, HER2 IHC, together with other markers, assists in the more precise histological classification of the carcinoma. Additionally, the presence of HER2 expression can be noted in the pathology report as relevant information for the oncologist in advanced-stage disease. As discussed, HER2 status correlates with molecular variants: strongly positive tumors are most often in the luminal group, whereas a lack of HER2 is characteristic of basal and double-negative classes. In the future, if HER2-targeted therapy enters clinical practice in bladder cancer, routine IHC assessment of HER2 would acquire diagnostic significance as well (similar to testing for PD-L1 or FGFR mutations in certain patients). At present, however, there are no official recommendations from international organizations (WHO, AJCC, ESMO, EAU) for mandatory HER2 testing in urothelial carcinoma—it is performed at the clinician’s discretion, mainly when there is a specific clinical need.

### 4.4. Prognostic Significance of HER2 Expression

Whether HER2 overexpression is an independent prognostic factor in bladder cancer has been investigated by numerous authors, but the results remain inconclusive. Some older studies suggest that HER2-positivity is associated with a worse course: for example, a shorter survival and earlier metastasis [9]. This seems logical given that aggressive variants like the micropapillary subtype (which is often HER2-positive) have a poor prognosis; in one study of micropapillary urothelial carcinoma (MPUC), patients with ERBB2 amplification had nearly a 3-fold higher risk of death from the disease compared to those without amplification [9]. Other analyses (Bolenz et al. 2010) have also reported that HER2 expression provides independent prognostic information in a multivariate model [10]. Conversely, many other studies have not found a statistically significant influence of HER2 on survival, especially when standard factors such as stage, grade, and therapy are accounted for [11]. In many cases, the HER2-positive tumors are luminal (some of which—papillary—have a relatively good prognosis) but also include aggressive morphologies (e.g., micropapillary), so the net effect on prognosis is neutralized. In summary, at present, HER2 is not established as an independent prognostic indicator in urothelial carcinoma. Rather, its impact is manifested indirectly, through the association with certain variants (for example, HER2(+)/luminal tumors vs. HER2(–)/basal tumors have different clinical courses). Further research on large cohorts is needed to isolate the effect of HER2 from other factors. There is evidence that HER2 acts as an oncogenic driver in a subset of bladder carcinoma cases [9], which supports the hypothesis that its overexpression might worsen clinical behavior, but definitive conclusions can be made only after prospective validation studies.

### 4.5. Therapeutic Significance and HER2-Targeted Therapy

Experience with anti-HER2 therapies in bladder cancer to date: Motivated by the success of anti-HER2 approaches in other carcinomas (breast, stomach), several clinical trials were conducted in urothelial carcinoma. For a long time, the results were disappointing, which hindered the incorporation of these therapies into the standard of care. For example, a phase II randomized trial by Oudard et al. (2015) in 61 patients with metastatic HER2-positive UCB compared chemotherapy (gemcitabine + platinum) with or without the addition of trastuzumab (a monoclonal anti-HER2 antibody) [11]. No significant difference in response or overall survival was observed between the two groups—the median OS was ~15 months in both (*p* = 0.68) [11]. Only in a subgroup analysis was a benefit noted for patients treated with cisplatin + trastuzumab (median OS 33 months) compared to carboplatin + trastuzumab (9.5 months) [11], suggesting that the choice of chemotherapy regimen has an influence. Overall, early trials with traditional anti-HER2 agents (monoclonal antibodies and small-molecule tyrosine kinase inhibitors like lapatinib) failed to demonstrate significant clinical benefits in bladder cancer. Potential reasons include the low frequency of suitable patients, the heterogeneity of HER2 status (including intratumoral heterogeneity and dynamic changes over time), and competition from effective chemo- and immunotherapies. As a result, no anti-HER2 therapy was approved for use in urothelial carcinoma in the past decade, and the routine testing of HER2 was not recommended by clinical guidelines (ESMO) outside of clinical trials [12].

New therapies—antibody–drug conjugates (ADCs): In the past few years, a resurgence of interest in HER2 as a therapeutic target has been observed, thanks to the development of ADC agents. These agents combine the specific binding of an anti-HER2 antibody with a potent cytotoxic “payload” that is delivered directly into tumor cells. Two ADC molecules in particular have shown promising results in urothelial carcinoma: disitamab vedotin (RC48) and trastuzumab deruxtecan (T-DXd). Disitamab vedotin is a humanized anti-HER2 antibody linked to a tubulin inhibitor (a conjugate with a molecule similar to monomethyl auristatin E). In a Chinese phase II trial in 107 patients with metastatic HER2-positive (IHC 2+/3+ or FISH+) urothelial carcinoma, this ADC achieved an objective response in 50.5% of patients (95% CI 40.6–60.3%) [13]. The median duration of response was ~6.9 months, and the safety profile was acceptable [13]. These results led to the approval of RC48 (disitamab vedotin) in China for treatment of HER2-positive advanced bladder cancer progressing after chemotherapy [14]. In parallel, global studies are investigating trastuzumab deruxtecan—an ADC combining trastuzumab with a topoisomerase I inhibitor [15]. The DESTINY-PanTumor02 trial (phase II, multicenter, 2024) included patients with urothelial carcinoma across various levels of HER2 expression. Primary results indicate that T-DXd produces clinically meaningful responses even in patients with IHC 2+/ heterogeneous HER2 expression [16,17,18]. The overall response rate for all solid tumors in the study was ~37% [16], and, in the bladder cancer subgroup, a median progression-free survival of ~7.0 months and overall survival of ~12.8 months were reported (data presented at ASCO GU 2025). These outcomes are comparable to or better than standard second-line chemo- or immunotherapy. Importantly, T-DXd has demonstrated activity even in cases with lower HER2 expression, potentially expanding the pool of patients who could benefit [18]. This aligns with recent trends in breast cancer, where the concept of a “HER2-low” therapeutic category was introduced, and T-DXd showed efficacy in that group. The final results from DESTINY and similar studies are anticipated to lead to new indications—for example, as of 2025, international guidelines (EAU) are already discussing the possibility of using trastuzumab deruxtecan in HER2-positive urothelial carcinomas, especially after the exhaustion of standard options.

Current guidelines and future perspectives: To date, standard targeted therapy for advanced urothelial carcinoma is directed mainly at other biomarkers—e.g., FGFR3 (erdafitinib) in tumors with FGFR3 mutations/fusions, as well as immunotherapy with checkpoint inhibitors (e.g., anti-PD-1/PD-L1) in appropriate patients. HER2-directed therapies are still not part of the routine algorithm. According to current ESMO and NCCN guidelines, routine testing of HER2 is not recommended in bladder cancer, and patients with HER2-positive tumors are encouraged to enroll in clinical trials [8]. However, this situation will likely change soon if ADC therapies continue to demonstrate efficacy. Variant histologies also affect the therapeutic approach—for example, in the micropapillary subtype [19], the guidelines (EAU, ESMO) recommend a more aggressive approach (early cystectomy, possibly neoadjuvant chemotherapy), but so far they do not specifically address HER2-targeting in this subtype. As further evidence accumulates, personalized therapy according to HER2 status may become a reality in urothelial carcinoma.

In the near future, the integration of HER2 status into the individualization of bladder carcinoma treatment is expected. For example, a patient with a luminal, HER2 3+ invasive tumor, after the exhaustion of standard therapies, could be directed to treatment with disitamab vedotin or trastuzumab deruxtecan through early access programs. The advent of these new agents will likely necessitate a change in the diagnostic workflow—if the presence of HER2 overexpression provides an additional line of therapy, then routine HER2 testing (similar to checking for FGFR mutations or PD-L1 status) would be justified for all patients with advanced urothelial carcinoma. Regulatory authorities (EMA, FDA) as of 2025 are closely monitoring developments—disitamab vedotin is under evaluation outside of China, and international phase III trials for trastuzumab deruxtecan are underway. In this dynamic context, a multidisciplinary approach is key—pathologists, oncologists, and urologists must jointly interpret HER2 results in the context of the specific tumor subtype and clinical situation, with a view to optimally planning treatment for each patient.

## 5. Conclusions

HER2 is an important biomarker with heterogeneous expression in urothelial carcinoma of the bladder. The present study showed that the frequency and level of HER2 expression vary substantially among different histopathological subtypes and molecular variants. Tumors with luminal differentiation—in particular, papillary and micropapillary—demonstrate a high frequency of HER2 overexpression, whereas basal tumors (urothelial carcinomas with squamous differentiation and sarcomatoid carcinomas) rarely express HER2. These differences reflect the underlying tumor biology and have potential diagnostic and prognostic significance. Although HER2 is not yet definitively established as an independent prognostic factor for bladder cancer, its expression often accompanies more aggressive phenotypes and may delineate subgroups at higher risk (for example, BCG-refractory superficial carcinomas or micropapillary invasive forms). The greatest significance of HER2 is as a therapeutic target. After unconvincing results with trastuzumab in the past, today the new generation of ADC agents (e.g., disitamab vedotin, trastuzumab deruxtecan) offers hope for effective HER2-directed therapy even in patients with low expression levels. The initial clinical successes of these agents highlight the possibility that stratification by HER2 status could improve personalized care in advanced urothelial carcinoma. In the future, results from ongoing studies are expected to lead to updates in clinical guidelines and the integration of HER2 as a criterion in therapeutic decision-making.

## Figures and Tables

**Table 1:** HER2 expression in the main histological subtypes of urothelial carcinoma.

Histological Subtype	Number of Cases (*n*)	HER2 3+	HER2 2+
Urothelial carcinoma (NOS)	4	0%	25%
Papillary urothelial carcinoma	27	44%	26%
Micropapillary subtype	4	75%	25%
Plasmacytoid subtype	3	33%	33%
UC with squamous differentiation	17	0%	0%
UC with glandular differentiation	3	0%	33%
Nested subtype	2	50%	0%
Sarcomatoid subtype	5	0%	0%
Small cell carcinoma	5	0%	0%

**Table 2:** HER2 status in IHC-based molecular profiling of MIBC in the studied cohort (*n* = 100).

Molecular Variant	Number of Cases (*n*)	HER2 3+ (%)	HER2 2+ (%)
Luminal Papillary	24 (24%)	33%	38%
Luminal Unstable	16 (16%)	56%	31%
Luminal Non-Specifiend (LumNS)	10 (10%)	50%	30%
Basal/Squamous	33 (33%)	0%	3%
BasoLuminal (Double-positive)	9 (9%)	33%	22%
NE-like	6 (6%)	0%	0%
Stroma-rich	2 (2%)	0%	0%
Total	100 (100%)	-	-

**Table 3:** Distribution by HER2 immunohistochemical (IHC) status.

HER2 IHC Category	Number of Cases (*n*)	Percentage (%)
0 (Negative)	31	31%
0+ (Ultra-low)	9	9%
1+ (Weak)	13	13%
2+ (Moderate)	22	22%
3+ (Strong)	25	25%
Total	100	100%

## Data Availability

The data that support the findings of this study are available from the corresponding author upon reasonable request.

## Abbreviations

The following abbreviations are used in this manuscript:

BC	Bladder carcinoma
MIBC	Muscle-invasive bladder carcinoma
UC	Urothelial carcinoma
HER2	Human Epidermal Growth Factor Receptor 2
IHC	Immunohistochemistry
TURB	Transurethral resection
RC	Radical cystectomy
FGFR	Fibroblast growth factor receptor 3
FISH/CISH	Fluorescence/chromogenic in situ hybridization
ADC	Antibody–drug conjugate
ASCO/CAP	American society of clinical oncology/College of American pathologists
EAU	European association of urology
ESMO	European society for medical oncology
NCCN	National comprehensive cancer network
